# Tracking the Invisible War: Automated Profiling of Multidrug-Resistant Pathogens in a Tertiary Care Teaching Hospital in Central India

**DOI:** 10.7759/cureus.85778

**Published:** 2025-06-11

**Authors:** Smita V Mohod, Dilip S Gedam, Arya L Rajan, Ravindra K Khadse, Riya John

**Affiliations:** 1 Microbiology, Indira Gandhi Government Medical College & Hospital, Nagpur, IND

**Keywords:** antimicrobial resistance, automated systems, bacterial identification, minimum inhibitory concentration, multidrug resistant organisms, vitek 2 compact

## Abstract

Background

Automated systems such as VITEK® 2 Compact have revolutionized microbial diagnostics by offering rapid identification and antimicrobial susceptibility testing (AST). This study aimed to evaluate the spectrum of bacterial and fungal isolates and their resistance profiles using the VITEK 2 system.

Material and methods

A retrospective cross-sectional analysis was conducted over one year (January 2024 to December 2024) in the Department of Microbiology of a tertiary care hospital in Central India. The present study included only those clinical specimens that were initially processed using conventional methods but proved difficult to identify based on biochemical reactions alone. These included blood, sputum, wound swab, pus, cervicovaginal swab, endotracheal aspirate, pleural fluid, bronchoalveolar lavage, stool, corneal scraping, and cerebrospinal fluid. Such samples were subsequently subjected to identification by the VITEK 2 Compact system to ensure rapid and accurate results. The resistance patterns of Gram-negative organisms including *Enterobacterales* and non-fermenters, gram-positive cocci, and yeasts were analyzed. These findings were entered into the Microsoft Excel Version 2010. Statistical analysis was carried out using SPSS Version 20 for Windows package (IBM Corp., Armonk, NY, USA). Observed association of multidrug resistant (MDR) isolates from ICU with observed multidrug resistance from non-ICU was tested by calculating the p-value using the chi-square test (p-value of 0.00055, i.e., p < 0.05 was considered significant).

Results

Out of 284 isolates, *Klebsiella pneumoniae* 35 (12%), *Pseudomonas aeruginosa* 24 (8%), *Staphylococcus aureus* 33 (11%), and *Candida albicans* 14 (5%) were predominant. Isolated organisms were found more in the ICU, 195 (69%), than non-ICU, 89 (31%), setting. The proportion of MDR isolates is significantly higher in the ICU (92.82%, 181/195) compared to the non-ICU setting (78.65%, 70/89). High resistance was noted among *Enterobacterales* against β-lactams (100%) and fluoroquinolones (87.5%). Non-fermenters such as *Acinetobacter baumannii* showed 100% resistance to multiple drugs, indicating pan-drug resistance in some strains. Among gram-positive organisms, penicillin, erythromycin, levofloxacin, ciprofloxacin, and chloramphenicol were tested for *Enterococcus faecalis. Enterococcus faecalis* showed 100% resistance to penicillin, erythromycin, and chloramphenicol. Yeasts exhibited varied resistance, with *Candida tropicalis *and *Cryptococcus laurentii* showing higher resistance to fluconazole, 4 (57%) and 1 (100%), respectively.

Conclusion

The study reveals a significant rising occurrence of multidrug-resistant organisms, particularly in critical care areas. The VITEK 2 Compact system enabled rapid and precise identification of resistance profiles, including rare and highly resistant strains. Its use is crucial for timely, targeted therapy and reinforces the need for robust diagnostic and antimicrobial stewardship practices.

## Introduction

Antimicrobial-resistant (AMR) infection has been ranked as the third leading cause of death after cardiovascular diseases [1]. It takes many years to develop a new antibiotic, but bacteria can become resistant in just a few months. This growing gap between discovery and resistance poses a serious challenge to effective treatment and public health. Accurate and rapid detection of resistance to antimicrobial drugs and subsequent appropriate antimicrobial treatment, combined with antimicrobial stewardship, are essential for controlling the emergence and spread of AMR [2]. The VITEK® systems from BioMérieux are widely employed in clinical microbiology laboratories globally. In our government teaching hospital, Indira Gandhi Government Medical College & Hospital, Nagpur, Maharashtra, India, routine microbial identification and antimicrobial susceptibility testing (AST) are primarily conducted using conventional methods due to financial constraints. Although several automated diagnostic systems such as VITEK 2, MALDI-TOF MS, BacT/ALERT, and molecular platforms are available across various healthcare settings in India, our institute is currently equipped only with the VITEK 2 Compact system and the BacT/ALERT system for bacterial identification and blood culture processing, respectively. Due to limited funding and high patient load, it is not feasible to perform automated testing for all clinical samples. VITEK 2 is selectively used in our laboratory, particularly for samples that yield inconclusive or ambiguous results through conventional biochemical identification or show doubtful antimicrobial susceptibility patterns on Muller-Hinton agar. These systems play a crucial role in species identification and AST for various clinical isolates. VITEK Advanced Expert System (AES) is designed to analyze AST results by utilizing a well-established knowledge base encompassing around 100 species and 20,000 MIC ranges. This allows the system to identify over 2,300 phenotypic antimicrobial resistances [3].

This study was initiated to overcome the limitations of conventional biochemical methods, which often fail to accurately or rapidly identify certain bacterial and fungal pathogens encountered in clinical practice. This is particularly critical in tertiary care hospitals, where timely diagnosis is essential for effective patient management, especially in intensive care settings. The use of the VITEK 2 Compact system enables automated and expedited identification and susceptibility testing, thereby improving diagnostic accuracy and reducing reporting time. By analyzing isolates from both ICU and non-ICU areas, the study aims to provide a comprehensive overview of microbial distribution and resistance trends. Such data are pivotal for optimizing empirical therapy, strengthening infection control strategies, and supporting antimicrobial stewardship initiatives within resource-constrained healthcare environments.

The primary objectives of the study were to identify bacterial and fungal pathogens isolated from clinical specimens using the VITEK 2 Compact system and to analyze the antimicrobial resistance patterns of the identified isolates through automated susceptibility testing. The secondary objectives were to compare the distribution of isolates obtained from ICU and non-ICU settings and to evaluate the diagnostic benefit of VITEK 2 Compact in detecting rare or biochemically inconclusive organisms that are challenging to identify by conventional methods.

## Materials and methods

A retrospective cross-sectional study was conducted over a period of one year, from January 2024 to December 2024, in the Department of Microbiology at a tertiary care teaching hospital in Nagpur, Maharashtra, India

A total of 284 clinical specimens including blood, respiratory secretions, pus, cervicovaginal swabs, stool, and sterile body fluids were processed under strict aseptic precautions. Each specimen was inoculated on appropriate culture media (blood agar, MacConkey agar, Sabouraud dextrose agar, etc.) and incubated under suitable atmospheric conditions (aerobic at 35-37°C) for 18-48 hours, following standard microbiological procedures.

Colonies demonstrating significant growth based on morphological characteristics such as colony size, pigmentation, hemolysis, and consistency with the specimen type were considered potentially pathogenic. Selection was further supported by the patient's clinical presentation and provisional diagnosis. These significant colonies were subcultured to obtain pure isolates and subjected to Gram staining for preliminary classification into Gram-positive cocci (GPC), Gram-negative bacilli, or yeast.

Microbial identification and AST were carried out using the VITEK 2 Compact system (Bio-Mérieux, France) following the manufacturer’s standardized protocol. A homogenous suspension equivalent to 0.50-0.63 McFarland standard was prepared for bacterial isolates, and a higher turbidity of 1.80-2.20 McFarland was used for yeast isolates, using a calibrated densitometer to ensure inoculum accuracy and reproducibility.

The following VITEK 2 cards were utilized according to Gram stain findings: VITEK 2 GP ID and AST-P628 cards for Gram-positive bacteria, VITEK 2 GN ID and AST-N405/N407 cards for Gram-negative bacteria, and VITEK 2 YST ID and AST-YS08 cards for yeast.

The system employs fluorescence-based kinetic measurements to detect metabolic activity and generate both organism identification and minimum inhibitory concentration (MIC) values. Susceptibility results were interpreted as sensitive (S), intermediate (I), or resistant (R) according to Clinical and Laboratory Standards Institute (CLSI) M100 guidelines [4].

Routine internal quality control and periodic validation of the VITEK 2 Compact system were conducted as per the manufacturer’s recommendations and institutional microbiology laboratory protocols. Commercially available ATCC quality control strains were employed at regular intervals to ensure consistent performance for both identification and AST across bacterial and fungal isolates. Only data generated during validated and quality-assured operational periods were included in this retrospective analysis.

The study included isolates that showed high confidence identification (97%-99%) by the VITEK 2 Compact system and were considered clinically significant based on their growth pattern and relevance to infection. Isolates were excluded if they were duplicates from the same patient and site, if they could not be identified by the VITEK 2 system, or if their susceptibility results were incomplete.

The MIC values and susceptibility interpretations were extracted directly from the VITEK reports. Resistance profiles were compiled and analyzed using Microsoft Excel Version 2010 (Microsoft Corp., Redmond, WA). Statistical analysis was carried out using SPSS Version 20 for Windows package (IBM Corp., Armonk, NY, USA). Results were expressed as percentages. Collected data were categorized into sample source, organism group, and percentage of MDR organisms according to ICU and non-ICU settings, and resistance pattern among isolated organisms was analysed using the VITEK 2 Compact machine. Association of MDR isolates in the ICU and the non-ICU setting was calculated using the chi-square test (p-value of 0.00055, i.e., p < 0.05 was considered significant).

## Results

**Figure 1:**
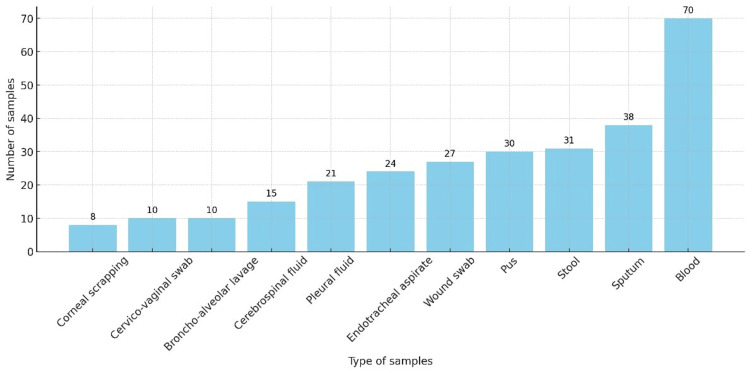
Samplewise distribution of isolates processed by VITEK 2 ( N= 284)

Figure 1 shows the distribution of 284 clinical isolates processed by VITEK 2. Blood samples were most common (n = 70), followed by sputum (38), while corneal scrapping showed the least number of samples (8).

**Figure 2:**
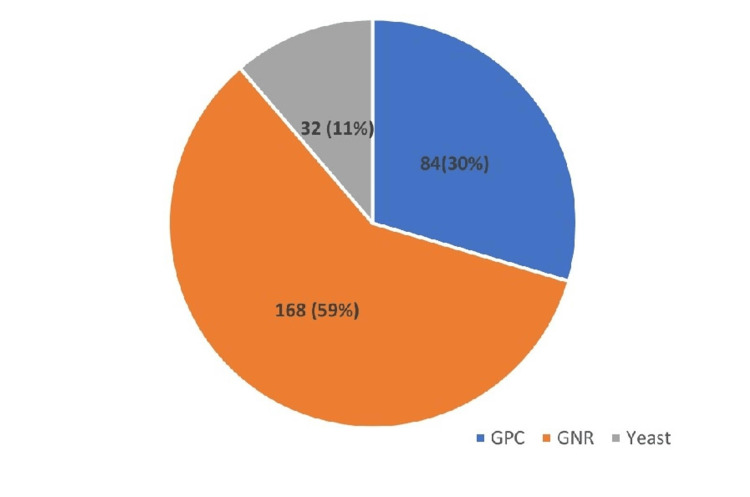
Types of organisms detected by VITEK 2 from the isolates (N=284) GNR, Gram-negative rod; GPC, Gram-positive cocci

The pie chart in Figure 2 illustrates the distribution of isolates processed by VITEK 2. Gram-negative rods (GNRs) were predominant, comprising 168 (59%) isolates. GPC accounted for 84 (30%) isolates, whereas yeasts made up 32 (11%) isolates.

**Figure 3:**
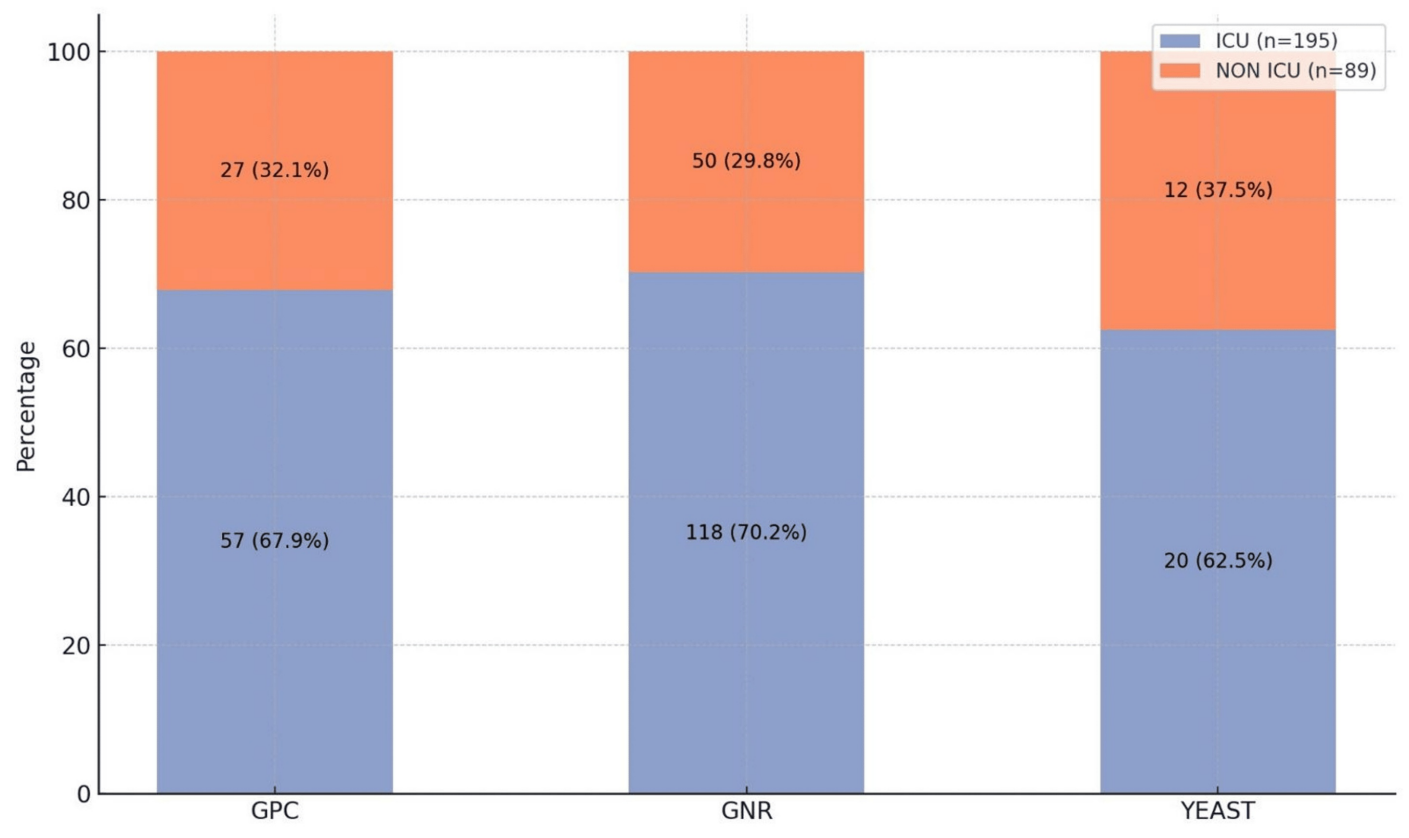
Distribution of isolates among ICU and non-ICU patients (N= 284) GNR, Gram-negative rod; GPC, Gram-positive cocci

As shown in Figure 3, among the 284 isolates, GNRs predominated (70.2% in ICU patients vs 29.8% in non-ICU patients), followed by GPC (67.9% in ICU patients vs 32.1% in non-ICU patients) and yeast (62.5% in ICU patients vs 37.5% in non-ICU patients). ICU samples consistently showed higher isolate distribution across all categories.

Table 1 shows that the proportion of multidrug-resistant (MDR) isolates was notably higher in ICU samples (92.82%) compared to non-ICU samples (78.65%), indicating a greater antimicrobial resistance burden in ICU settings.

**Table 1:** Percentage of MDR isolates according to ICU and non-ICU settings The p-value was calculated to compare the association between percentages of MDR in ICU and non-ICU settings using the chi-square test (p-value = 0.00055, i.e., p < 0.05). This indicates a statistically significant difference, suggesting that ICU isolates are more likely to be MDR than non-ICU isolates. MDR, multidrug resistance

Source of samples	Total isolates (N = 284)	Percentage of multidrug resistant isolates (n =251)
ICU	195	181 (92.82%)
Non-ICU	89	70 (78.65%)

All isolates showed high resistance to cephalosporins and carbapenems. *Klebsiella pneumoniae *and *Escherichia coli *were also resistant to aminoglycosides and fluoroquinolones. *Proteus*, *Providencia*, *Morganella*, and *Serratia *showed 100% resistance to most beta-lactams but retained sensitivity to aztreonam, azithromycin, and amikacin. Colistin resistance was not detected in any isolate (Table 2).

**Table 2:** Antimicrobial resistance pattern of Enterobacterales isolates on VITEK 2 Note: 0 denotes no resistance found or 100% sensitivity, while (-) denotes drug is not tested for the organism IR, intrinsic resistance

Antimicrobials	*Klebsiella pneumoniae *(n =35)	*Escherichia coli* (n = 22)	*Escherichia hermannii *(n =7)	*Enterobacter cloacae* (n = 9)	*Serratia marcescens* (n = 5)	*Providentia stuarti* (n = 4)	*Pantoea *sp. (n = 6)	*Vibrio cholerae* (n = 12)
Amoxycillin- clavulanic acid	26 (75%)	11 (50%)	0	IR	IR	IR	-	12 (100%)
Cefazolin	35 (100%)	11 (50%)	7 (100%)	IR	IR	IR	6 (100%)	-
Ceftriaxone	35 (100%)	11 (50%)	7 (100%)	9 (100%)	5 (100%)	4 (100%)	6 (100%)	-
Cefuroxime	35 (100%)	11 (50%)	7 (100%)	9 (100%)	IR	4 (100%)	6 (100%)	-
Ceftazidime	35 (100%)	11 (50%)	7 (100%)	9 (100%)	5 (100%)	4 (100%)	6 (100%)	12 (100%)
Cefixime	35 (100%)	11 (50%)	7 (100%)	9 (100%)	5 (100%)	4 (100%)	6 (100%)	-
Cefoxitin	35 (100%)	11 (50%)	7 (100%)	IR	IR	4 (100%)	6 (100%)	-
Cefotaxime	35 (100%)	11 (50%)	7 (100%)	9 (100%)	5 (100%)	4 (100%)	6 (100%)	-
Cefepime	35 (100%)	11 (50%)	7 (100%)	0	5 (100%)	4 (100%)	6 (100%)	-
Piperacillin- tazobactam	26 (75%)	11 (50%)	0	0	3 (67%)	0	0	12 (100%)
Ampicillin/sulbactam	35 (100%)	11 (50%)	5 (67%)	IR	IR	0	0	-
Imipenem	35 (100%)	11 (50%)	7 (100%)	9 (100%)	5 (100%)	4 (100%)	6 (100%)	6 (50%)
Meropenem	35 (100%)	11 (50%)	7 (100%)	0	0	4 (100%)	6 (100%)	6 (50%)
Ertapenem	35 (100%)	11 (50%)	7 (100%)	0	0	4 (100%)	6 (100%)	-
Gentamicin	26 (75%)	11 (50%)	7 (100%)	0	0	IR	4 (100%)	6 (50%)
Amikacin	26 (75%)	11 (50%)	7 (100%)	0	2 (33%)	4 (100%)	6 (100%)	6 (50%)
Levofloxacin	30 (87.5%)	11 (50%)	7 (100%)	6 (67%)	5 (100%)	4 (100%)	6 (100%)	0
Ciprofloxacin	30 (87.5%)	11 (50%)	7 (100%)	6 (67%)	5 (100%)	4 (100%)	6 (100%)	0
Tetracycline	-	-	-	-	-	IR	-	-
Doxycycline	-	-	-	-	-	IR	-	-
Ampicillin	IR	-	-	IR	IR	IR	-	6 (50%)
Azithromycin	-	--	-	-	-	IR	-	6 (50%)
Cotrimoxazole	35 (100%)	11 (50%)	0	0	0	4 (100%)	6 (100%)	12 (100%)
Aztreonam	35 (100%)	11 (50%)	0	9 (100%)	5 (100%)	4 (100%)	6 (100%)	-
Tigecycline	-	-	-	-	-	IR	-	-
Colistin	0	0	0	0	IR	IR	0	-

*Sphingomonas paucimobilis* (all four from blood) showed 100% resistance to all tested antibiotics (Table 3). *Ochrobactrum anthropi* (three from blood and two from pleural fluid) exhibited 100% resistance to aminoglycosides, fluoroquinolones, cotrimoxazole, and aztreonam. *Stenotrophomonas maltophilia *(three from blood and one from endotracheal tube) showed intrinsic resistance to multiple antibiotics including beta-lactams, aminoglycosides, and aztreonam, but 0% resistance to levofloxacin, ciprofloxacin, doxycycline, ceftazidime, cefepime, and cotrimoxazole. *Elizabethkingia meningoseptica *(three from blood) showed 100% resistance to all tested drugs except colistin (0% resistance).

**Table 3:** Antimicrobial resistance pattern of miscellaneous gram-negative isolates on VITEK 2 Note: 0 denotes no resistance found or 100% sensitivity, while (-) denotes drug is not tested for the organism. IR, intrinsic resistance

Antimicrobials	*Sphingomonas paucimobilis *(n = 4)	*Ochrobactrum anthropi* (n = 5)	*Stenotrophomonas maltophilia *(n = 4)	*Elizabethkingia meningoseptica* (n = 3)
Amoxycillin- clavulanic acid	4 (100%)	0	IR	3 (100%)
Cefazolin	4 (100%)	5 (100%)	-	3 (100%)
Ceftriaxone	4 (100%)	5 (100%)	IR	3 (100%)
Cefuroxime	4 (100%)	5 (100%)	-	3 (100%)
Ceftazidime	4 (100%)	5 (100%)	4 (100%)	3 (100%)
Cefixime	4 (100%)	5 (100%)	-	3 (100%)
Cefoxitin	4 (100%)	5 (100%)	4 (100%)	3 (100%)
Cefotaxime	4 (100%)	5 (100%)	IR	3 (100%)
Cefepime	4 (100%)	5 (100%)	4 (100%)	3 (100%)
Piperacillin – tazobactam	4 (100%)	5 (100%)	IR	3 (100%)
Ampicillin/sulbactam	-	-	IR	-
Imipenem	4 (100%)	0	IR	3 (100%)
Meropenem	4 (100%)	0	IR	3 (100%)
Ertapenem	4 (100%)	0	IR	3 (100%)
Gentamicin	4 (100%)	0	IR	3 (100%)
Amikacin	4 (100%)	0	IR	3 (100%)
Levofloxacin	4 (100%)	0	4 (100%)	3 (100%)
Ciprofloxacin	4 (100%)	0	4 (100%)	3 (100%)
Tetracycline	-	-	IR	-
Doxycycline		-	4 (100%)	-
Ampicillin	-	-	IR	-
Azithromycin	-	-	-	--
Cotrimoxazole	4 (100%)	0	4 (100%)	3 (100%)
Aztreonam	4 (100%)	0	IR	3 (100%)
Tigecycline	-	-	0	-
Colistin	0	0	0	0

All four non-fermenters - *Pseudomonas aeruginosa*, *P. fluorescens*, *Acinetobacter baumannii*, and *A. lwoffii* - exhibited high resistance to beta-lactams (including ceftazidime), aminoglycosides, fluoroquinolones, and cotrimoxazole (Table 4). Carbapenem resistance was complete in *P. aeruginosa *and *A. lwoffii *and partial in *P. fluorescens *(imipenem 33%, meropenem 67%) and *A. baumannii *(imipenem 50%, meropenem 70%). All isolates showed intrinsic resistance to multiple agents. Notably, colistin remained uniformly effective (0% resistance) across all species.

**Table 4:** Antimicrobial resistance pattern of non-fermenter isolates by VITEK 2 Note: 0 denotes no resistance found or 100% sensitivity, while (-) denotes drug is not tested for the organism. IR, intrinsic resistance

Antimicrobials	*Pseudomonas aeruginosa* (n = 24)	*Pseudomonas fluorescens* (n = 13)	*Acinetobacter baumannii *(n = 10)	*Acinetobacter lwoffii *(n = 5)
Amoxycillin- clavulanic acid	IR	IR	IR	IR
Cefazolin	IR	IR	10 (100%)	5 (100%)
Ceftriaxone	IR	IR	10 (100%)	5 (100%)
Cefuroxime	IR	IR	10 (100%)	5 (100%)
Ceftazidime	12 (100%)	12 (100%)	10 (100%)	5 (100%)
Cefixime	IR	IR	10 (100%)	5 (100%)
Cefoxitin	IR	IR	10 (100%)	5 (100%)
Cefotaxime	IR	IR	10 (100%)	5 (100%)
Cefepime	0	0	10 (100%)	5 (100%)
Piperacillin – tazobactam	0	4 (33%)	10 (100%)	5 (100%)
Ampicillin/sulbactam	IR	IR	10 (100%)	5 (100%)
Imipenem	12 (100%)	4 (33%)	5 (50%)	5 (100%)
Meropenem	12 (100%)	9 (67%)	7 (70%)	5 (100%)
Ertapenem	IR	IR	IR	IR
Gentamicin	12 (100%)	0	10 (100%)	5 (100%)
Amikacin	12 (100%)	0	10 (100%)	5 (100%)
Levofloxacin	12 (100%)	9 (67%)	10 (100%)	5 (100%)
Ciprofloxacin	12 (100%)	9 (67%)	10 (100%)	5 (100%)
Tetracycline	IR	IR	-	-
Doxycycline	IR	IR	-	-
Ampicillin	IR	IR	IR	IR
Azithromycin	-	-	-	--
Cotrimoxazole	12 (100%)	13 (100%)	4 (40%)	3 (67%)
Aztreonam	12 (100%)	4 (33%)	IR	IR
Tigecycline	IR	IR	-	-
Chloramphenicol	IR	IR	IR	IR
Colistin	0	0	0	0

GPC showed high resistance in *Enterococcus faecalis *to multiple drugs (100%), while *S. aureus *showed 67% resistance to key antibiotics (Table 5). Methicillin-resistance *S. aureus *(MRSA) strains were fully resistant to cefoxitin, *E. faecium *showed moderate resistance (33-67%), and *Kocuria rosea* from corneal scrapping was resistant to most of the tested drugs.

**Table 5:** Antimicrobial resistance pattern of Gram-positive cocci isolates on VITEK 2 Note: 0 denotes no resistance found or 100% sensitivity, while (-) denotes drug is not tested for the organism. IR, intrinsic resistance

Antimicrobials	*Staphylococcus aureus *(n = 33)	Methicillin-resistance *Staphylococcus aureus* (n = 15)	*Enterococcus faecalis* (n = 22)	*Enterococcus faecium *(n = 13)	*Kocuria rosea* (n = 1)
Penicillin	22 (67%)	9 (60%)	22 (100%)	4 (33%)	0
Cefoxitin	0	15 (100%)	IR	IR	1 (100%)
Erythromycin	0	0	22 (100%)	4 (33%)	1 (100%)
Clindamycin	0	0	IR	IR	0
Gentamycin	0	0	IR	IR	1 (100%)
Amikacin	0	0	IR	IR	1 (100%)
Levofloxacin	0	0	0	9 (67%)	0
Ciprofloxacin	0	0	0	9 (67%)	0
Tetracycline	0	0	-	-	-
Doxycycline	0	0	-	-	-
Chloramphenicol	22 (67%)	9 (60%)	15 (67%)	4 (33%)	0
Ampicillin	-	-	22 (100%)	4 (33%)	-
High-level gentamycin	-	-	22 (100%)	4 (33%)	-
Vancomycin	-	-	0	0	-
Linezolid	22 (67%)	0	0	0	0
Tigecycline	-	-	-	-	0
Cotrimoxazole	11 (33%)	9 (60%)	IR	IR	-

*Candida tropicalis* showed the highest fluconazole resistance (57%), followed by *C. krusei* and *C. parapsilosis* (50% each) and *Cryptococcus laurentii*, which showed 100% resistance to all antifungals (Table 6). Moderate resistance to amphotericin B and flucytosine was noted across isolates.

**Table 6:** Antifungal resistance pattern of yeast isolates on VITEK 2 Note: 0 denotes no resistance found or 100% sensitivity

Antimicrobials	*Candida albicans* (n = 14)	*Candida tropicalis* (n = 7)	*Candida krusei *(n = 6)	*Candida parapsilosis* (n = 4)	*Cryptococcus laurentii* (n = 1)
Fluconazole	6 (39%)	4 (57%)	3 (50%)	2 (50%)	1 (100%)
Voriconazole	5 (36%)	3 (43%)	2 (33%)	2 (50%)	1 (100%)
Caspofungin	0	0	0	0	1 (100%)
Micafungin	0	0	0	0	1 (100%)
Amphotericin B	4 (28%)	2 (29%)	2 (33%)	1 (25%)	1 (100%)
Flucytosine	3 (21%)	1 (14%)	1 (17%)	1 (25%)	1 (100%)

## Discussion

This study presents a comprehensive evaluation of antimicrobial resistance patterns across diverse bacterial and fungal pathogens using the VITEK 2 Compact system. The findings provide critical insights into emerging resistance trends, particularly in ICU settings. Our study revealed a significantly higher prevalence of MDR among ICU isolates (92.8%) compared to non-ICU isolates (78.7%), aligning with global trends that highlight the ICU as a hotspot for antimicrobial resistance. The MDR rate observed in our ICU cohort closely parallels the findings of Siwakoti et al. [5], who reported an 88% MDR rate in ICU settings, thereby reinforcing the notion that critically ill patients are particularly vulnerable to resistant infections. In contrast, Hamadalneel et al. [6] reported a notably lower ICU MDR rate of 39.3%, suggesting that variations in antimicrobial stewardship practices, infection control measures, and pathogen distribution may account for these disparities. The elevated MDR burden in our ICU population is likely attributable to the frequent use of broad-spectrum antibiotics, increased reliance on invasive devices, and the severity of illness among these patients.

Although our study did not evaluate clinical outcomes, the high MDR prevalence in ICU settings underscores its potential impact on patient prognosis. These findings highlight the urgent need for robust antimicrobial stewardship programs, continuous surveillance for resistance mechanisms (such as ESBL [extended spectrum beta-lactamase] and carbapenemase production), and strict infection prevention strategies. Evidence from other settings indicates that targeted interventions, including early identification and isolation of MDR cases, can significantly curb the transmission of resistant organisms in ICUs.

Among *Enterobacterales*, *K. pneumoniae *and *E. coli* demonstrated 100% resistance to third-generation cephalosporins and high resistance to fluoroquinolones and >85% resistance to ceftazidime and ciprofloxacin in *K. pneumoniae *and *E. coli *in a tertiary hospital in Nepal, attributing the surge to ESBL and AmpC β-lactamase production [7]. Bora et al. also reported 86.3% and 87.6% prevalence of MDR in *E. coli *and *K. pneumoniae *isolates, respectively, in Northeast India [8]. Non-fermenters, particularly *A. baumannii*, exhibited complete resistance to imipenem, meropenem, and cephalosporins, confirming the presence of extensively drug-resistant strains. Similar findings were reported by Vijayakumar et al., where ICU-derived *A. baumannii *isolates showed 100% resistance to carbapenems, associated with the presence of blaOXA-23 [9]. *Pseudomonas aeruginosa *demonstrated extensive resistance to β-lactams and aminoglycosides, consistent with the study by Hancock and Speert [10].

Rare non-fermentative organisms such as *S. maltophilia*, *O. anthropi*, and *E. meningoseptica* showed intrinsic and acquired resistance to multiple drug classes. *Stenotrophomonas maltophilia *is typically intrinsically resistant to multiple and broad-spectrum antibiotic agents [11] *Elizabethkingia meningoseptica *is usually multi-resistant to antibiotics typically prescribed for Gram-negative bacterial infections, including most extended-spectrum β-lactams [12], emphasizing the need for precise identification and individualized therapy.

*Pantoea *species have also been isolated from clinical specimens. *Pantoea agglomerans *is the most commonly isolated species within genus in humans, resulting in soft tissue or bone/joint infections following penetrating trauma by vegetation [13].

The VITEK 2 Compact system proved highly effective in rapid identification, particularly excelling in detecting rare isolates such as *Pantoea*, *Ochrobactrum*, and *Kocuria*, often missed by conventional methods. Its advanced algorithm and automated analysis improve diagnostic turnaround time and pathogen detection. However, due to inherent biochemical variability in these uncommon organisms, occasional misidentification may arise, as reported in previous studies by Soutar et al. and Kate et al. noted that biochemical anomalies in rare organisms could occasionally lead to misidentification, necessitating confirmatory methods for accuracy [14,15]. This highlights the importance of using supplementary automated techniques, particularly when identifying rare or clinically significant isolates to ensure diagnostic precision and appropriate antimicrobial therapy.

Linezolid retained 100% activity against MRSA and Enterococci, affirming its ongoing role in managing resistant Gram-positive infections. Nevertheless, emerging resistance has been noted in Northern Europe, as highlighted by Misiakou et al., necessitating cautious use and robust antimicrobial stewardship programs [16].

Among Enterococci, both *E. faecalis *and *E. faecium* were 100% sensitive to vancomycin and linezolid in the present study. Among Enterococci, both *E. faecalis *and *E. faecium *were 100% sensitive to vancomycin and linezolid in the present study, while Salem-Bekhit et al. reported an emerging trend of vancomycin-resistant *Enterococcus* isolates, showing 8.7% resistance to vancomycin in a South Indian tertiary care center [17]. This observation differs from our results. Vancomycin resistance among *Enterococcus* isolates is a major problem in most of the western world, especially in the United States, where, according to the National Nosocomial Infections Surveillance System (NNIS) data, more than 28% of all nosocomial *Enterococcal* strains are resistant to vancomycin [18]. This variation emphasizes the importance of local epidemiological surveillance.

Fungal isolates, notably *Candida albicans*, *C. tropicalis*, and *C. krusei*, showed moderate to high resistance to azoles. Magobo et al. reported 68% fluconazole resistance in *C. parapsilosis*, especially among ICU patients [19]. *Candida krusei* showed intrinsic resistance to fluconazole and partial cross-resistance to voriconazole, consistent with the observations by Pfaller et al., who emphasized the limited efficacy of azoles against non-albicans *Candida* infections [20].

Cryptococcal meningitis is the most common cause of fungal meningoencephalitis worldwide and the most common cause of fungal central nervous system infection in the immunosuppressed host [21]. In the present study, *C. laurentii* exhibited complete resistance to fluconazole, voriconazole, and amphotericin B, a rare phenomenon but one previously documented among immunocompromised hosts. Averbuch et al. reported a similar fluconazole resistance pattern in a cancer patient [22].

Overall, the study reinforces the significance of automated systems in antimicrobial resistance surveillance while highlighting the critical need for continuous local antibiogram generation, integrated into national AMR control policies, to ensure rational antimicrobial therapy and strengthen stewardship initiatives.

This study has certain limitations. Molecular techniques were not employed to confirm the underlying resistance mechanisms, which may have limited the precision in characterizing antimicrobial resistance. Additionally, patient clinical outcomes were not evaluated, restricting the ability to correlate microbiological findings with clinical relevance. This study does not include a comparative analysis with other alternative methods. Furthermore, anaerobic and fastidious organisms were excluded from the analysis due to the limitations of the identification system used.

## Conclusions

This study demonstrates the practical utility of automated diagnostic systems, particularly the VITEK 2 Compact, in strengthening the surveillance of multidrug-resistant organisms. By analyzing 284 clinical isolates over one year, we observed a notable predominance of resistant Gram-negative pathogens, especially *K. pneumoniae* and *P. aeruginosa*, highlighting the narrowing spectrum of effective antimicrobials in the ICU setting in a tertiary care hospital, Nagpur, Central India.

The adoption of automation redefined routine culture-based diagnostics into a more proactive and time-sensitive approach. The ability to rapidly detect resistance patterns offers critical support to antimicrobial stewardship programs by enabling early initiation of targeted therapy and minimizing empirical misuse. However, the true value of such technology lies in integrating these laboratory findings into broader clinical and public health strategies.

Based on our results, we recommend routine reporting of automated susceptibility data to institutional antimicrobial stewardship teams and periodic submission to national AMR surveillance networks. Establishing a structured feedback mechanism between diagnostic laboratories and clinicians will enhance therapeutic decisions and optimize patient outcomes. The integration of automated systems should be viewed not merely as a laboratory upgrade but as an essential component of national efforts to contain antimicrobial resistance.
